# Generation Paths of Major Road Accidents Based on Fuzzy-Set Qualitative Comparative Analysis

**DOI:** 10.3390/ijerph192113761

**Published:** 2022-10-22

**Authors:** Yu Lei, Guirong Zhang, Shan Lu, Jiahuan Qian

**Affiliations:** 1 School of Public Administration, Central South University, Changsha 410083, China; 2 Center for Social Stability Risk Assessment, Central South University, Changsha 410083, China

**Keywords:** emergency management, major road accidents, influencing factors, generation mechanism, qualitative comparative analysis

## Abstract

In the process of continuously promoting safety management, major road accidents have become a key obstacle to improving overall road safety. The analysis of the overall road accidents hides the characteristics and laws of major road accidents. To clarify the causes of major road accidents, an analysis framework of “individual-vehicle-environment-management” is presented based on a literature review. Considering the interaction of the above variables, the fuzz-set qualitative comparative analysis (fsQCA) was used to explore the generating paths of major road accidents based on 42 road accidents. The work shows that: (1) Major road accidents are caused due to the interactive coupling of “individual-vehicle-environment-management” elements. Major road accidents can occur with normal driving behaviors or sufficient response and rescue capabilities. (2) General road accidents and relatively major road accidents are more likely to occur in the presence of driving behavior errors, favorable road facilities, and sufficient response and rescue capabilities. Moreover, major road accidents are more likely to occur due to large vehicles with adverse vehicle performances. (3) There are three path modes and five condition configurations in major road accidents, namely individual-vehicle-management induced, individual-vehicle-environment induced, and vehicle induced mode. This work enriches the accident causation mode from a new configuration perspective and explains which variable combinations lead to the occurrence of major road accidents. Clarification of the differences between general accidents and major accidents will help to accurately predict and restrain the development of major road accidents.

## 1. Introduction

Despite a considerable investment of manpower, material, and financial resources, road accidents are still the major cause of death and injury. Road accidents inflict a severe negative impact on the physical and mental health of the victim and their families, thus reducing their quality of life and work performance [1]. It is noteworthy that major road accidents are regarded as an important issue in safety management in various countries around the world. Specifically, compared to ordinary accidents (general road accidents refers to accidents that cause less than three deaths, serious injuries to less than 10 persons, or direct economic losses of less than 10 million yuan [2]), major road accidents (accidents causing the death of more than 10 persons, or causing serious injury to more than 50 persons, or causing direct economic losses of more than 50 million Yuan [2]) are a huge obstacle in improving overall road safety (the major road accidents in this paper refer to the above road accidents). Major road accidents have serious consequences. In addition, major road accidents happen from time to time. Management aimed at reducing major road accidents has not reached the ideal state. The question of what conditions cause major road accidents is worth considering deeply. By analyzing the development process of road accidents, we can better understand the regularity and internal causes of major road accidents to explore their governance mechanism. Therefore, studies and analyses of major road accidents are urgently needed and are challenging.

There are various works in the literature that explain the occurrence and factors leading to road accidents, reveal the essential rules of road accidents, and explain their severity [3]. The major causes of road accidents are presented below.

(1) Individual factors. The mechanism of “perception-judge-operation” [4] states that the main cause of road accidents is the improper handling of a vehicle in dangerous situations. The individual factors leading to road accidents include behavioral characteristics [5], physical and psychological states, and demographic characteristics [6]. First, the most important factor that influences the occurrence of road accidents and deaths are driving behaviors, such as over speeding, fatigue, failure to use protective devices [7], and lack of familiarity with local driving rules [5]. Over speeding is the most common cause reported in all recent and past road accidents [5]. Second, the physical and mental status are associated with road accidents. The use of alcohol or drugs significantly impacts road safety [6]. The meta-analysis shows a significant association between car crashes and drowsy driving [8]. Third, driving habits are interconnected with living standards and social culture, as well as gender, age, education level, and income [7]. The structural equation model has been used to demonstrate that a driver’s gender and age are associated with road accidents [3]. The analysis shows that male drivers have a higher accident rate in comparison to female drivers [7]. Similarly, younger and older drivers are involved in more accidents, as compared to middle-aged drivers [9].
(2) Vehicle factors. The vehicle characteristics also influence the risk of injury in automobile accidents [6]. The safety conditions of vehicles, such as vehicle overload, availability of compulsory third-party insurance of vehicle, whether the vehicle is a commercial vehicle [7], and the condition of tires [5] affect the occurrence of road accidents severity. In addition, there is evidence that the severity of accidents is more affected by vehicle volume, as compared to speed [10].
(3) Road factors. The number of fatal accidents is dependent on road conditions, such as types of road surface [3], road length [11], horizontal curvature of road [12], road friction, average daily traffic flow, average daily percentage of trucks, and number of overpasses per mile [12]. The negative binomial model shows that large traffic volume, over speeding, narrow lane widths, higher number of lanes, urban road segments, narrow shoulder widths, and reduced middle widths increase the probability of accidents [9]. Additionally, the number of casualties is higher in areas where the connectivity and accessibility are not good [13]. The collisions involving multiple vehicles during lane-changing are more likely to occur on wet roads, while the rear-end collisions are more likely to occur on dry roads during the daytime [10].
(4) Weather factors. Adverse weather conditions (such as precipitation, fog, dust, rain, snow, and high temperatures) and driving errors were identified as major contributing factors in approximately two-thirds of road accidents [5]. The adverse weather events affect visibility and reduce road friction, thus increasing the chance of road accidents. Similarly, glare on sunny days is also detrimental to road safety [14]. The lighting conditions are also identified as variables that affect severity [15]. Few researchers believe that street lighting conditions, weather conditions, visibility, occurrence of accidents on weekends or public holidays, time of day, season, and accident year [7] affect the risk of road accidents. However, the influence of weather factors is controversial. The precipitation generally leads to an increase in the frequency of accidents, there does not seem to be an effect on the severity of accidents [16]. At the same time, weather conditions or accident timings do not seem to affect the risk of injury [6].
(5) Management factors. The traffic rules and legislation can reduce or control the rate of traffic violations, thereby reducing the incidence of serious injuries and deaths [7]. Pre-licensure and post-licensure education improves self-perception and slightly decreases traffic offenses among all the age groups [17]. Through interviews and questionnaires, it was found that the road-safety climate affects the road safety in communities [18], which is an important embodiment of road safety management.

Thus, existing studies have reached a basic consensus on the causes of road accidents by mainly focusing on individual factors, vehicle factors, road factors, weather factors, and management factors. However, it is noteworthy that the existing research on the causes of major road accidents have still not addressed the following issues clearly.

(1) The existing literature does not pay enough attention to the generation mechanism of the major road accidents. Statistics of large samples (such as structural equations [3]) cannot distinguish the characteristics of the different accidents and cover up the unique laws of major road accidents. Analyzing the frequency and likelihood of accidents by analyzing the negative binomial model techniques from road and traffic characteristics [9] does not take enough account of the severity of accidents. The generation mechanism of major road accidents may be different from general and relatively major road accidents. Therefore, a detailed study is required to understand the nature and necessary conditions of major road accidents. This study will help in improving overall road safety situations.
(2) The research regarding the interaction of the causes of major road accidents has not been conducted in-depth. The existing modes usually describe the relationship between the consequences of road accidents and the corresponding independent influencing factors. However, they fail to reflect the complex interactions among the influencing factors. The existing research has not presented any specific causal combination of elements and modes for major road accidents. The traffic system is a complex and dynamic system comprising individuals, vehicles, roads, weather, management, and other factors. The coupling of these factors is the cause of the instability of the system. No single factor seems to be the key factor in determining the severity of an accident, but a single factor can act as a catalyst barrier in conjunction with other factors to impact the severity of injury [6]. “Linear combinations of multiple variables can effectively explain accident characteristics and modes [10]”, and “factors such as road geometry, driver characteristics and vehicle types interact in complex ways to influence the scale of traffic accident scale [3]”. In this work, we try to establish a causal coupling theory of road accidents that emphasizes the causal coupling relationship.
(3) The influence of weather conditions on the consequences of road accidents is controversial. On the one hand, weather conditions are not significantly associated with accident severity [7]. Weather conditions do not seem to affect the severity of road accidents [6]. Severe weather conditions reduce the risk of traffic violations and have no significant effect on accident severity [7]. On the other hand, weather conditions are considered to be an important factor in determining the fatality rate of road accidents in the United States [19]. Weather conditions can affect road safety by affecting drivers and road systems [12]. In addition to driving errors, adverse weather conditions were identified as the main cause of road accidents [5].
(4) The influence of vehicle types on the consequences of road accidents is uncertain. Trucks have a significantly higher risk of traffic violations and accident severity, and enhanced lorry vehicle safety and overload status check are critical to reducing road violations and accident severity rates [7]. However, trucks are an insignificant factor in the severity of road accident injuries in the absence of traffic violations behaviors [7]. Vehicle types sometimes affect the consequences of road accidents, and sometimes have no effect. Thus, what kind of complex causal relationship between vehicle types and major road accidents has not been answered.

In response to the above questions, this work used 42 road accidents and fuzzy-set qualitative comparative analysis (fsQCA) from the perspective of configuration to analyze the generating paths and governance mechanism of major road accidents in order to improve road safety. The paper is structured as follows: The Section 2 concerns the Research Design, which mainly includes the introduction of Research Methods and Ideas, Case Selection, and Variable Design. The Section 3 provides the analysis of the Empirical Results, which mainly includes the Single Factor Necessity and Sufficiency Analysis, Configuration Analysis of Sufficient Conditions, and Generation Mechanism of Major Road Accidents. Section 4 and Section 5 provide the Discussions and Conclusions.

## 2. Research Design

### 2.1. Research Methods and Ideas

Fuzzy set qualitative comparative analysis (fsQCA) is based on set theory and Boolean algebra to determine whether the condition configuration constitutes a sufficient subset for the occurrence of outcome variables [20]. It is dedicated to exploring how multiple condition variables collectively affect outcome variables by discussing the affiliations [21] between the sets. The QCA method regards the case as a whole, which his composed of causative conditions, so as to find the causal relationship between the matching patterns of different conditions and the results. QCA is a relatively new research method [22], and its specific application steps are as follows (See Ragin, 2008 for more information):

(1) Determine the outcome variables and condition variables of the study, formulate the calibration rules and assign values to the variables based on case facts and theoretical knowledge. The degree of affiliation of variables is defined as a fuzzy set between non-subordinate (0) and complete subordination (1) [23].
(2) Establish a truth value table and list the score combination of condition variables and result variables of each case [24].
(3) Import the truth table into the fsQCA software for calculation (see the software manual for the operation process (Ragin, 2017 [25])), and simplify the relationship [26] between the conditions and results through the Boolean algebra to obtain the combination of necessary conditions and sufficient conditions of the result.

FsQCA3.0 software has a special manual, more specific operation processes see the software manual (Ragin, 2017 [25]).

The fsQCA has the following adaptability to the research question of this paper. (1) Due to the difficulty in obtaining official data of road accidents, it is not possible to obtain a large number of samples. The research object samples of this paper are small, so regression analysis is not applicable. A total of 42 road accidents in China from 2011 to 2020 are collected through the authoritative website. The fsQCA enables us to analyze small and medium-sized samples of road accidents. The fsQCA is suitable for analyzing the concurrent situation of multiple conditions, as it can analyze the coupling relationships between different factors and reveal the combined paths of major road accidents. (2) Based on the existing research presented in the literature, the generation of major road accidents is a typical multi-cause and one-effect problem, which contains the causal logic of many factors interacting with each other. The fsQCA is suitable for analyzing the concurrent situation of multiple conditions, as it can analyze the coupling relationships between different factors and reveal the combined paths of major road accident generation. Therefore, this work uses the fsQCA method to explore the generation mechanism of major road accidents. Since it is difficult to define and measure the cases (accidents) that have not occurred in the real world, this work transforms the study from the research of relatively non-transformation to the problem of a high degree of severity by focusing on exploring the differences between the generation mechanism and conditional causes of major accidents (including major accidents and particularly major accidents in this paper) and general accidents (including general accidents and relatively major accidents in this paper).

### 2.2. Case Selection

The number of sample selections will affect the analysis results. Choose the most appropriate number of cases, we studied what was closer to the real world. The fsQCA method has a unique advantage in the study of the number of small cases (cases of 10 or 15 or less) and medium-scale samples (cases of 15 to 50) [21]. Therefore, medium-scale samples, ranging from 15 to 50, are mostly selected for research.

The sources of road accident cases in this work mainly include several aspects. First, in order to ensure the authority and stability of the cases, we search the official websites of the Ministry of Emergency Management of the People’s Republic of China for the investigation reports of major road accidents between 2011 and 2020. We collected nine particularly major accidents and supplemented them with the investigation report of major road accidents published on the safety management website. Based on the safety production management website, the most recent 13 major road accidents were collected, a total of 22 accidents were established and the experimental group was established. Because accidents in recent years can provide a reference for road safety management. Second, in order to ensure the heterogeneity of variables and to expand the differences between cases to identify different pathways or combinations of factors that produce the results [26], the relatively major accidents and general accidents are studied as a non-set of major accidents. According to the rules of case collection of qualitative comparative analysis, the control group should account for half of the total cases collected. The research cases are selected according to the principles of reference, typicality, and homogeneity. In order to improve the temporal homogeneity of the experimental group, 9 relatively major road accidents with a similar time to particularly major road accidents were selected from the safety production management website as the control group. Finally, 11 general road accidents with a similar time to major road accidents were selected from the safety production management website as the control group, and 20 control groups were established.

Therefore, 42 road accidents were included in the case database (see Appendix A Table A1). Among them, 13 major accidents and 9 particularly major accidents were regarded as the research objects, accounting for 52.4% of the total case database. The 11 general accidents and 9 relatively major accidents were considered as a set of general accidents and relatively major accidents, accounting for 47.6% of the total case database.

### 2.3. Variable Design

In this work, we select the major road accidents as the output variable. The selection process of condition variables is as follows.

**Figure 1:**
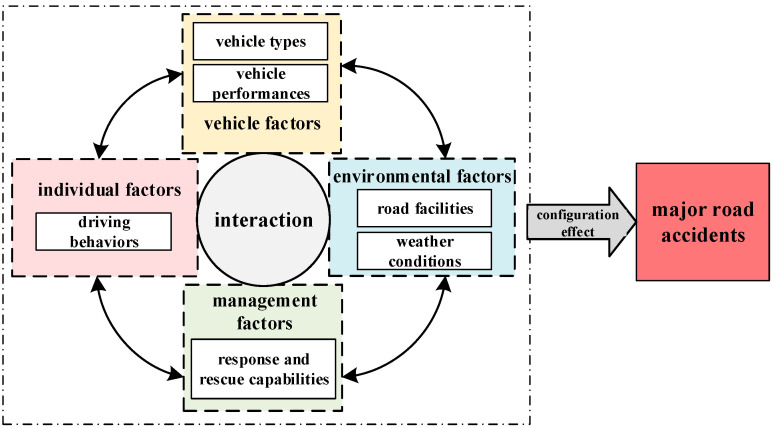
The analysis mode of the configuration effect for major road accidents.

On the one hand, the main influencing factors of Figure 1 are obtained by existing research, including individual factors, vehicle factors, environment factors, and management factors. Road factors are the key ways to prevent road accidents in many accident investigation reports. This paper considers road facilities and weather conditions as sub-variables of environmental factors and analyzes the combination path of road facilities and five other factors that leads to result variables.

On the other hand, the measurement of the main influencing factors needs to be achieved through sub-variables. The variables recommended by fsQCA are selected between three and seven [21] and a sub-variable that has a great influence on the outcome variables when measuring the condition variables are selected. Therefore, due to the number of variables limited by the research method, the variable selection in Figure 1 mainly considers importance, representativeness, and measurability, rather than comprehensiveness. This measurement method is designed to achieve a more scientific choice to measure the sub-variables that mainly affect the outcome variable. Based on the literature review and case knowledge, as well as full consideration of the variables operability [27], we select driving behaviors, vehicle types, vehicle performances, road facilities, weather conditions, and response and rescue capabilities as the condition variables. The “individual-vehicle-environment-management” research framework of this paper is established, as presented in Figure 1. Based on the main influencing factors obtained by the existing research, analyze the interaction and coupling effects between them (see Figure 1). In the figure, arrows indicate the interaction between the main influencing factors. In the solid line box, the sub-variables that affect the factors are used to measure the main influencing factors.

The original data is transformed into membership scores by calibration [28]. The assignment process of result variables and condition variables is presented in Table 1 (1) Based on the severity of the accidents, the general, relatively major, major, and particularly major accidents are assigned 0, 0.33, 0.67 and 1. (2) Similarly, we assign a value to the vehicle involved in the accidents based on its mass. (3) The emergency response capability for an accident is largely reflected in the time required for the restoration of unilateral traffic on the road or the rescue and evacuation of injured or casualties. The response and rescue capabilities (continuous variable) is converted into a four-valued fuzzy-set based on the quartile [29]. The lower quartile (25%) of the data is regarded as the completely unaffiliated calibration point, the median (50%) is regarded as the intersection point, and the upper quartile (75%) is regarded as the fully affiliated calibration point. This fuzzy-set is used as the measurement method of emergency response capability. (4) The assignment rules of other dichotomous variables are detailed in Table 1. In addition, in order to avoid the situation that the case is difficult to be classified and not included due to the intersection, the value of its membership degree of 0.5 is set to 0.51 [30].

This paper draws a social network analysis chart to visually present the cause of accidents. Social network analysis is an analytical method developed from the graph theory to measure the network structure. Social network analysis is usually used to analyze key nodes from structural perspectives, interactions between different nodes, and overall network structures (such as in Reference [31]), and display the relationship between individuals, and overall, in a visual form. The central degree refers to the degree that one node in the network is related to all other nodes. The larger the central degree of a node, the more important the node is in the network.

**Figure 2:**
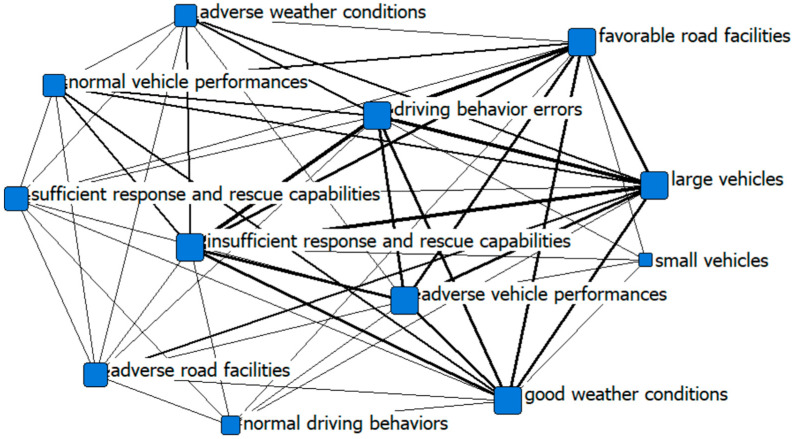
The social network analysis of major road accidents.

**Figure 3:**
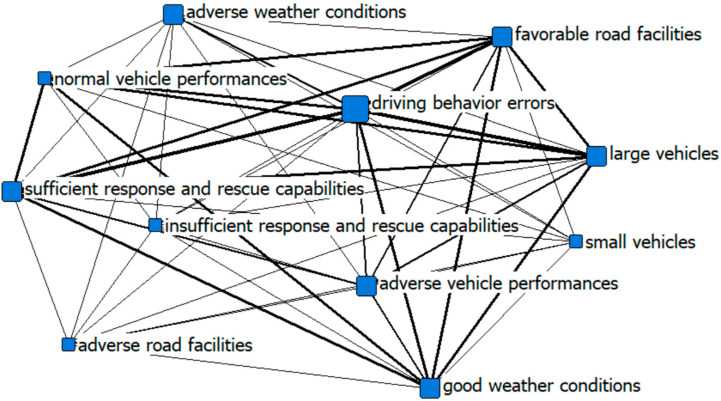
The social network analysis of general road accidents.

We transform the case data into a truth table based on the operational definition and assignment rules of the variables. By considering the membership degree of 0.5 as the boundary and the conditional configuration as the accident chain, we draw a social network analysis diagram to visually present the 42 accident causal combinations, as shown in Figure 2 and Figure 3. From the perspective of network structure indicators, it shows the differences between the causes of particularly major accidents and general accidents. The size of the point in Figure 2 and Figure 3 is adjusted based on the degree, and the thickness of the connection is adjusted according to the strength of the association. The three major causes of major road accidents are vehicle types, driving behaviors, and response and rescue capabilities based on the rank of the network degree centrality, while the three major causes of general road accidents include driving behaviors, favorable road facilities, and vehicle types. At the same time, it is obvious that there are differences in the number of nodes, the size of nodes, and the closeness of connections between major accidents and general accidents.

In order to further quantify the difference between the two, the frequency of cases is set to one, the consistency threshold is set to 0.8, and the PRI consistency threshold is set to 0.7 [28]. The single factor necessity and sufficiency are further calculated to measure the importance of condition variables.

## 3. Empirical Analysis

### 3.1. Single Factor Necessity and Sufficiency Analysis

The single factor necessity and sufficiency analysis are performed using fsQCA3.0 software. The corresponding computation results are presented in Table 2. In the necessity test, the consistency of a condition variable is greater than 0.9, which is a necessary condition for the outcome variables [32]. When the consistency of a condition variable is in the interval [0.8,0.9], which is the sufficient condition of the outcome variables [33]. Based on this, driving behavior errors and large vehicles are necessary conditions for major road accidents. Driving behavior errors are a necessary condition for general and relatively major road accidents, and large vehicles and favorable road facilities are sufficient conditions. Next, we analyze the configuration of condition variables to obtain more information regarding the generation path of major road accidents.

### 3.2. Configuration Analysis of Sufficient Conditions

In this work, we use FsQCA3.0 software to analyze the simplified and intermediate solutions of major road accidents. The corresponding computation results of configuration analysis are presented in Table 3. The consistency of the obtained solution should be greater than 0.8, and the coverage should be greater than 0.5 [32]. The research shows that the solution to major road accidents is considered to meet the requirements of consistency and coverage [32]. The path is a combination of condition variables implemented by the result variables. There are five configuration paths for major road accident generation [28]. The consistency of the five configuration paths is 0.814, indicating that 81.4% of the cases in the combination of the five configuration paths are major road accidents. The coverage is 0.530, indicating that the five interpretation paths can explain 53.0% of the occurrence of major road accidents. In addition, the core conditions and edge conditions are distinguished based on the union of simplified and intermediate solutions [28]. The condition variables obtained from the union of simplified and intermediate solutions are the core conditions. It is noteworthy that the core conditions significantly influence the outcome variables. The other conditions are marginal and have a little influence on the outcome variables. The “core condition exists” as a large dot, and the “edge condition exists“ as a small dot in Table 3.

Based on the configuration of sufficient conditions, we explore the characteristics of condition variables in major road accidents.

(1) These five configuration paths conform to the characteristics of different paths and the same destination. The condition variables of each configuration path are multiple and concurrent. The optimal interpretation power configuration ranking, based on the original coverage, is Path 1 > Path 3 > Path 2 > Path 5 > Path 4.
(2) Generally, it is assumed that the driving behavior errors play a major role in road accidents. The road accidents occur when there are errors in the driving behaviors and vehicle performances are adverse, such as Paths 1, 2, and 3. However, Path 4 and Path 5 show that the normal driving behaviors, coupled with other factors, may lead to major road accidents as well. The Paths 4 and 5 show that the major road accidents may occur either due to the driving behavior errors or adverse vehicle performances. This is because the major road accidents are caused by the interactive coupling of individual factors, vehicle factors, environmental factors, and management factors.
(3) Satisfaction or dissatisfaction with condition variables can lead to major road accidents. Small vehicles (Path 1), sufficient response and rescue capabilities (Path 4), and normal vehicle performances (Path 3) lead to major road accidents.

### 3.3. Generation Mechanism of Major Road Accidents

Refine and name the generation modes of major road accidents according to the characteristics of existing conditions in the paths [32]. The five paths are summarized based on three modes, including individual-vehicle-management induced major road accidents (Path 1), individual-vehicle-environment induced major road accidents (Path 2 and Path 3), and vehicle induced major road accidents (Path 4 and Path 5). In addition, we also analyze these paths in detail.

(1) Individual-vehicle-management induced major road accidents: Configuration Path 1 typically represents the individual-vehicle-management induced major road accidents. Here, the core conditions include adverse vehicle performances, good weather conditions, and sufficient response and rescue capabilities, combined with driving behavior errors and favorable road facilities as edge conditions. This shows that in the accidents with favorable road facilities and good weather conditions, the influence of vehicle types is not high, and the major road accidents are caused by the driving behavior errors, adverse vehicle performances, and insufficient response and rescue capabilities. The individual-vehicle-management induced typical accident includes the Lhasa, Tibet “8.9” particularly major road accident in 2014.
(2) Individual-vehicle-management induced major road accidents: Configuration Path 2 typically represents the individual-vehicle-management induced major road accidents. Here, the core conditions include large vehicles, adverse road facilities, and adverse weather conditions, combined with driving behavior errors and adverse vehicle performances as edge conditions. This type of accident does not require high or low response and rescue capabilities, mainly due to driving behavior errors, large vehicles, adverse vehicle performances, adverse road facilities, and adverse weather conditions that lead to major road accidents. The example of individual-vehicle-environment induced typical accident is Xi’an “11.13”, which was major road accident in 2018.
(3) Vehicle induced major road accidents: Configuration Path 4 typically represents vehicle-induced major road accidents. Here, the core condition includes normal driving behaviors, and the marginal conditions include large vehicles, adverse vehicle performances, favorable road facilities, good weather conditions, and sufficient response and rescue capabilities. This shows that, under normal driving behaviors, favorable road facilities, good weather conditions, and sufficient response and rescue capabilities, major road accidents are mainly caused by large vehicles and adverse vehicle performances. The example of the vehicle-induced accident is the Shaanxi Xianyang “5.15”, particularly in the case of the major road accident in 2015.

According to the aforementioned analysis, it is evident that there are three path modes of major road accidents. First, the individual-vehicle-management induced path mode with adverse vehicle performances, good weather conditions, and insufficient response and rescue capabilities as the core elements, leads to major road accidents. Second, the individual-vehicle-environment induced path mode with large vehicles, adverse road facilities, and adverse weather conditions as the main factors, lead to major road accidents. Third, the vehicle induced path mode with normal driving behaviors as the main reason. These three modes show that the generation of major road accidents require contribution from multiple factors.

## 4. Discussion

The major contributions of this work are presented below.

(1) Responding to the lack of attention in the existing literature on the generation mechanism of major road accidents. Our work reveals the facts covered by the large sample research. The necessary conditions are generally different between general accidents with relatively major accidents. Based on the comparative analysis of a single factor, the differences between sufficient and necessary conditions of major accidents and general accidents are obtained in a fine-grained manner. General and relatively major road accidents are more likely to occur in the presence of driving behavior errors, favorable road facilities, and sufficient response and rescue capabilities. The major road accidents are more likely to occur due to large vehicles and adverse vehicle performances.
(2) Responding to the lack of consideration for the interaction of the causes of major road accidents. As opposed to univariate analysis, which does not consider variable interaction under large sample statistics, we use the Boolean algebra operation of the set theory to understand which combination of variables leads to major road accidents from the perspective of configuration. This paper proposes a new idea for configuration based on the coupling analysis framework of the “individual-vehicle-environment-management” system. Based on road accident cases, this work considers the interaction of 4 major causes of road accidents and explores five major road accident generation paths. It can better understand the possible path of major road accidents and realize the promotion from the research of influencing factors to the research of generation mechanism. Based on conditional configuration analysis, it is proved that a “single factor can act as a catalyst or barrier when combined with other factors affecting the severity of injuries [6]. The five paths break through the gap between theory and practice, accurately and completely reflect the influencing factors and generation mechanism of major road accidents and help to formulate appropriate methods and policies to improve road safety.
(3) Responding to controversy over the impact of weather conditions on the consequences of road accidents. We agree that weather conditions are important factors affecting major road accidents [19]. In the results of the single factor necessity and sufficiency for the major road accidents, the consistency of good weather conditions is high (value of 0.710). It partly refutes the view that weather conditions and the severity of accidents are not significantly related [7]. More importantly, this paper argues that weather conditions play different roles in different configuration paths of major road accidents. Paths 2 and path 3 demonstrate that adverse weather and wet roads can help reduce the scale of accidents [3]. Paths 1, 4, and 5 prove that major road accidents can occur in good weather conditions. This study refutes the view that weather conditions reduce major road accidents, and we believe that there is a path leading to major road accidents, whether a certain condition in “individual-vehicle-environment-management” is met or not.
(4) Responding to a certain degree of uncertainty in the impact of vehicle types on the consequences of road accidents. Different from the view that trucks are insignificant factors in the severity of road accident injuries in the absence of traffic violation behaviors [7], this paper proposes five different causal paths between vehicle types and major road accidents. In different coupling situations of the vehicle types and the other five conditions, different forms of vehicle types will lead to major road accidents. This paper provides evidence for the impact of vehicle types on accident severity (Path 4 and 5) in major road accidents without traffic violation behaviors, thus enhancing the safety of trucks is crucial to reducing accident severity. Different from previous studies on the impact of coupling between vehicle types and driving behaviors, this paper extends to the interaction of “individual-vehicle-environment-management”, and further considers the impact of different coupling forms of variables on major road accidents.

However, limited by the size of the research sample, the availability of case data acquisition sets the frequency threshold to 1 in the fsQCA. The generalization of the research may be affected to a certain extent. In future research, we will try to expand the scope of the samples and combine the quantitative analysis (such as variable interaction analysis) to verify the research conclusions. Further research can also be performed to explore the configuration paths of general and major road accidents. Secondly, driving behavior errors can be further subdivided based on the severity of the consequences, such as different penalties for drunk driving and inattentiveness. Finally, the proposed work can be further extended to study other accident generation mechanisms to enrich the accident causation mode.

## 5. Conclusions

In this work, we used fsQCA to study 42 major road accidents. We clarified the influencing factors and generation mechanism of major road accidents and tried to answer the three questions mentioned in the Introduction. (1) What is the cause of major road accidents? Major road accidents are caused by the coupling of various factors in the framework of “individual-vehicle-environment-management”. At the same time, there are differences in the necessary and sufficient conditions for general and major road accidents. Among these factors, driving behavior errors (the consistency of its single factor is 0.903) and large vehicles (the consistency of its single factor is 0.967) are the necessary conditions for major road accidents. Driving behavior errors (the consistency of its single factor is one) are necessary conditions for general accidents and relatively major accidents, and large vehicles (the consistency of its single factor is 0.890) and favorable road facilities (the consistency of its single factor is 0.891) are sufficient conditions. (2) How do the causes of major road accidents interact? From the perspective of conditional configuration, it is observed that no matter whether a single individual, vehicle, environment, and management factors are satisfied, there are major modes of road accidents. (3) What are the generating paths of major road accidents? The five generating paths of major road accidents are classified into three path modes, including individual-vehicle-management induced, individual-vehicle-environment induced, and vehicle induced. The individual-vehicle-management induced type is more likely to cause major road accidents. The configuration ranking of the preferred explanatory power based on the original coverage is path 1 (its value is 0.282) > path 3 (Its value is 0.107) > path 2 (Its value is 0.081) > path 5 (Its value is 0.048) > path 4 (Its value is 0.046).

In order to reduce major road accidents, the three configurations of individual-vehicle-management induced, individual-vehicle-environment induced, and vehicle-induced are disintegrated according to the necessary conditions and configuration paths. First, reduce the errors of driving behavior, and focus on the investigation and treatment of large vehicles and their performances. Second, the path of major road accidents can be determined according to the configuration path combined with the actual situation. Weather conditions should be concerned during vehicle driving, and the road facilities and rescue capabilities can be constructed so as to crack the combination elements in a targeted way and build a comprehensive system of “individual-vehicle-environment-management” of major road accidents.

## Figures and Tables

**Table 1:** The operational definition and assignment of variables.

Types	Variable Name	Sub-Variables	Sub-Variable Statistics Rules	Assignment	Assignment Support
Outcome variables	Severity of road accidents	-	Particularly major accident	1	Assign values based on quartiles
		-	Major accident	0.67	
		-	Relatively major accident	0.33	
		-	General accident	0	
Condition variables	Individual factors	*X*_1_ driving behaviors	Overloading, over speeding, improper operations, fatigue driving, drunk driving, drug driving, driving without a license, inconsistent with the permitted driving types, inattentiveness, physical disability, etc.	1	Investigation report facts
			driving behaviors in compliance with road traffic regulations	0	
	Vehicle factors	*X*_2_ vehicle types	Largest vehicle involved in the accident is a large, extra-large bus or heavy goods truck	1	Assign values based on quartiles
			Largest vehicle involved in the accident is a mid-size bus or truck	0.67	
			Largest vehicle involved in the accident is a light bus or truck	0.33	
			Otherwise	0	
		*X*_3_ vehicle performances	Bad braking, steering out of control, quality problems of parts, illegal modification, etc.	1	Investigation report facts
			Vehicle performances do not affect the accident occurrence	0	
	Environmental factors	*X*_4_ road facilities	Road is not equipped with one of the isolation belts, marking lines, signal lights, protective facilities, and warning signs, or a command post is set up in violation of regulations	1	Investigation report facts
			road protection facilities and signs have been properly installed	0	
		*X*_5_ weather conditions	Adverse weather (rain, snow, and fog), wet road surface, dim light, etc.	1	Investigation report facts
			Weather conditions do not affect the accident occurrence	0	
	Management factors	*X*_6_ response and rescue capabilities	Time required for one-way road recommencement or casualty evacuation after accident (hours)	1	Assign values based on Quartiles
				0.01	

**Table 2:** The necessity test of a single condition based on the fsQCA method.

Condition Variables	Outcome Variables	
	Consistency of Major Road Accidents	Consistency of General and Relatively Major Road Accidents
Driving behavior errors	0.903	1.000
Normal driving behaviors	0.097	0.000
Large vehicles	0.967	0.890
Small vehicles	0.113	0.188
Adverse vehicle performances	0.549	0.406
Normal vehicle performances	0.451	0.594
Adverse road facilities	0.226	0.109
Favorable road facilities	0.774	0.891
Adverse weather conditions	0.290	0.235
Good weather conditions	0.710	0.765
Insufficient response and rescue capabilities	0.749	0.343
Sufficient response and rescue capabilities	0.368	0.770

**Table 3:** The realization of the configuration of major road accidents by using fsQCA.

Accident Cause	Sub-Variable	Conditional Configuration of Major Road Accidents				
	Condition Variables	Path 1	Path 2	Path 3	Path 4	Path 5
		Individual-Vehicle-Management Induced	Individual-Vehicle-Environment Induced		Vehicle Induced	
Individual factors	Driving behaviors	●	●	●	Ⓧ	Ⓧ
Vehicle factors	Vehicle types	-	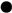	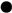	●	●
	Vehicle performances	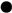	●	-	●	●
Environmental factors	Road facilities	⊗	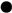	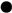	⊗	●
	Weather conditions	Ⓧ	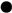	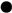	⊗	⊗
Management factors	Response and rescue capabilities	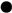	-	●	⊗	●
Original coverage		0.282	0.081	0.107	0.046	0.048
Unique coverage		0.282	0.047	0.073	0.046	0.048
Solution consistency		0.814				
Solution coverage		0.530				

(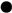; = core condition exists; Ⓧ = core condition missing; ● = edge conditions exists; ⊗ = edge condition missing; - indicates that the condition may or may not exist).

## Data Availability

Not applicable.

## Appendix A

**Table A1:** List of selected case database of road accidents that occurred in China between 2011 and 2020.

Serial Number	Types	Cases	Serial Number	Types	Cases
1	Particularly major	Changshen expressway Jiangsu Wuxi “9.28” particularly major road accident in 2019	23	Relatively major	Xingtai Town Xingzuo highway “2.27” relatively major road accident in 2018
2	Particularly major	Shaanxi Ankang Jingkun Expressway “8.10” particularly major road accident in 2017	24	Relatively major	Fuyang section of Chuxin expressway “11.15” relatively major road accident in 2017
3	Particularly major	Hunan Chenzhou Yifeng expressway “6.26” particularly major road accident in 2016	25	Relatively major	Tongxu Section of Daguang Expressway “10.2” relatively major road accident in 2017
4	Particularly major	Shaanxi Xianyang “5.15” particularly major road accident in 2015	26	Relatively major	Jiangyou city, Jiulingchang Town “7.23” relatively major road accident in 2017
5	Particularly major	Lhasa Tibet “8.9” particularly major road accident in 2014	27	Relatively major	Huangchuan Section of Shanghai-Shaanxi expressway “5.11” relatively major road accident in 2017
6	Particularly major	Hunan Shaoyang section of Hukun expressway “7.19” particularly major road deflagration accident of dangerous chemicals in 2014	28	Relatively major	Pingqiao section of national highway 312 “4.17” relatively major road accident in 2017
7	Particularly major	Baomao expressway Shaanxi Yan’an “8.26” particularly major road accident in 2012	29	Relatively major	Xuchang section of Yongdeng expressway “3.14” relatively major road accident in 2015
8	Particularly major	Binbao expressway Tianjin “10.7” particularly major road accident in 2011	30	Relatively major	Shaoyang city, Xinshao county, “7.27” relatively major road accident in 2014
9	Particularly major	Beijing-Zhuhai expressway Henan Xinyang “7.22” particularly major sleeper bus combustion accident in 2011	31	Relatively major	Mayang county “3.20” relatively major road accident in 2014
10	Major	Songyuan “10.4” major road accident in 2020	32	General	Dongguan City, Humen town “1.17” general road accident in 2019
11	Major	Xiangtan Huashi town “9.22” major road accident in 2019	33	General	Huangjiang town “11.24” general road accident in 2018
12	Major	Xi’an “11.13” major road accident in 2018	34	General	Chashan town “8.10” general road accident in 2018
13	Major	Lanlin section of the G75 Lanhai expressway “11.3” major road accident on the in 2018	35	General	Dalingshan town “6.21” general road accident in 2018
14	Major	Hengyang section of Beijing-Hong Kong-Macao expressway “6.29” major road accident in 2018	36	General	Shijie town “6.20” general road accident in 2018
15	Major	Ganzhou “2.20” major road accident in 2018	37	General	Fenggang town “5.21” general road accident in 2018
16	Major	Xinxiang section of Beijing-Hong Kong-Macao expressway “9·26” major road accident in 2017	38	General	Chang’an town “1.23” general road accident in 2018
17	Major	Zhangjiakou city Yu district 109 national highway “7.21” major road accident in 2017	39	General	Chang’an town “12.16” general road accident in 2017
18	Major	Longmen section of Guanghe expressway “7.6” major road accident in 2017	40	General	Dongguan city, Wanjiang street “12.10” general road accident in 2017
19	Major	Yicheng district, Zaozhuang city “10.13” major road accident in 2016	41	General	Shipai town “11.5” general road accident in 2017
20	Major	Jinji expressway “7.1” major road accident in 2016	42	General	Qiaotou town “10.2” general road accident in 2017
21	Major	Daguang expressway Xinyang Xin town “9.11” major road accident in 2015			
22	Major	Anyang Linzhou “3.2” major road accident in 2015			
